# Classification of intrusive thought patterns based on differences in the mechanisms of occurrence and persistence

**DOI:** 10.3389/fpsyt.2025.1520496

**Published:** 2025-03-20

**Authors:** Saki Hinuma, Hiroyoshi Ogishima, Hironori Shimada, Yuki Tanaka, Masumi Osao, Chihiro Moriishi, Shugo Obata

**Affiliations:** ^1^ Department of Clinical Psychology, Graduate School of Health and Welfare Sciences, International University of Health and Welfare, Tokyo, Japan; ^2^ Division of Information Science, Nara Institute of Science and Technology, Nara, Japan; ^3^ Faculty of Human Sciences, Waseda University, Saitama, Japan; ^4^ Faculty of Humanities, Wayo Women’s University, Chiba, Japan; ^5^ Yoyogi Sleep Disorder Center, Tokyo, Japan; ^6^ Human Informatics and Interaction Research Institute, The National Institute of Advanced Industrial Science and Technology (AIST), Ibaraki, Japan; ^7^ Department of Clinical Psychology, International University of Health and Welfare, Tokyo, Japan

**Keywords:** intrusive thoughts, co-clustering, obsessive-compulsive disorder (OCD), coping strategy, cognitive behavioral theory

## Abstract

**Introduction:**

Intrusive thoughts occurring independently of intention are symptoms of obsessive-compulsive disorders (OCD). However, they also appear in various other disorders, including substance use disorders, depression, post-traumatic stress disorder, and anxiety disorders, as well as in healthy individuals. Despite this, the diversity of intrusive thoughts remains largely unexplored. In this study, we aimed to (1) classify the factors causing intrusive thoughts as identified in previous research and (2) elucidate differences in the psychological states of intrusive thoughts.

**Methods:**

We investigated 298 participants over 20 years old using a questionnaire that includes scales such as “obsessive-compulsive belief,” “stress responses,” “thought suppression,” and “evaluation of intrusive thoughts.” To analyze data, we applied co-clustering, a machine-learning technique, to the data obtained from the investigation.

**Results:**

We identified three factors that affect the occurrence of intrusive thoughts: “Negative Evaluation of Intrusive Thoughts,” “Stress Responses,” and “Excessive Control of Intrusive Thoughts.” Furthermore, based on the scoring patterns of these three factors, participants were classified into five subtypes characterized by their degree of OCD tendencies. Further analysis revealed that the three factors could not be explained by OCD tendencies. Additionally, it was found that the five subtypes employed different coping strategies.

**Discussion:**

These findings suggest that intrusive thoughts cannot be fully explained solely by the degree of OCD tendencies, which could provide valuable insights into cognitive-behavioral support targeting the various psychological states associated with intrusive thoughts.

## 1 Introduction

Intrusive thoughts are defined as “thoughts that arise independently of intention and are difficult to control (1)”. These have mainly been recognized as a feature of obsessive-compulsive disorder (OCD). Nevertheless, it is also observed in various other disorders, including substance use disorders, depression, post-traumatic stress disorder, and anxiety disorders (2, 3). Furthermore, intrusive thoughts are commonly seen even in healthy individuals (4–7). Given this, examining why intrusive thoughts are broadly observed across diverse populations, including healthy individuals and patients with disorders other than OCD, could prove beneficial in treating OCD, which can be treatment-resistant and is known to co-occur with various other disorders. However, as previous research has largely focused on their relationship with OCD, there is still an insufficient understanding of these questions.

Generally, when a symptom is observed in a wide range of populations, it is essential to assume that diverse factors contribute to its occurrence and persistence. In this context, the cognitive-behavioral theory provides a valuable perspective. Based on this theory, intrusive thoughts are presumed to occur and are maintained by irrational cognitions and maladaptive behaviors and are exacerbated through interactions with environmental factors, such as stress (8–10). Within this framework, cognitive factors, such as “cognitive beliefs,” “cognitive evaluations,” and “thought suppression,” as well as environmental factors such as “stress states,” have been implicated in the occurrence of intrusive thoughts, alongside everyday behavioral factors such as “stress-coping strategies,” which are not necessarily specific to OCD (11–17). For example, a positive correlation exists between the occurrence of intrusive thoughts and stress. It has been suggested that a complex process underlies this relationship, in which increased stress triggers thought suppression and inhibits cognitive reappraisal (17). However, cognitive evaluation of events can also trigger stress responses. For instance, presumably having a complex causal relationship: evaluating intrusive thoughts as ego-dystonic can increase stress, and coping strategies for alleviating stress can lead to compulsive behaviors (11, 12).

Drawing from this evidence, clearly the mechanisms underlying intrusive thoughts are quite complex and their occurrence is not attributed to a single cause but rather to an interaction of various factors. Further, how these factors maintain intrusive thoughts in everyday contexts needs to be investigated. Although approaches that focus on the characteristics of intrusive thoughts within individual disorders acknowledge their presence across different conditions, they do not fully capture the interrelated factors that contribute to the occurrence and persistence of these thoughts (18). Consequently, there has been insufficient insight into why intrusive thoughts are observed across various populations beyond OCD.

Here, we attempted to gain a deeper understanding of the diversity of intrusive thoughts by investigating how the combination of factors, as examined within cognitive-behavioral theory, contributes to their occurrence and persistence of intrusive thoughts. To that end, we conducted a questionnaire-based analog survey targeting healthy individuals and analyzed the data using a data-driven method called co-clustering (19). Unlike traditional clustering methods (single-sided clustering), co-clustering can simultaneously classify both the similarity of the participants and the scales. For instance, when considering the relationship between the factors related to intrusive thought occurrence (i.e., obsessive beliefs, thought suppression, and stress responses), traditional clustering classifies participants based on the similarity of their scores. In contrast, co-clustering categorizes not only the participants, but also the factors contributing to the occurrence of intrusive thoughts. This approach also allows factors to be classified, such as obsessions occurring with thought suppression but not with stress, and participants are categorized into multiple subtypes based on the scoring patterns of these factors. That is, co-clustering allows for the exploration of which participant subtypes (participant clusters) have high scores for which factors influence the occurrence of intrusive thoughts (scale cluster),” while considering the relationships between these two factors.

In this study, we aimed to (1) clarify the differences among participant subtypes associated with intrusive thoughts by examining the complex co-occurrence relationships among the factors influencing the occurrence of intrusive thoughts according to cognitive-behavioral theories. Furthermore (2), we seek to elucidate the differences in factors that contribute to maintaining intrusive thoughts in each identified participant subtype, which will shed light on the varying adaptive strategies used in everyday contexts. Finally (3), by investigating the relationships among the factors associated with intrusive thoughts, participant subtypes, and obsessive-compulsive tendencies, we aimed to determine which aspects of intrusive thoughts show continuity with OCD. This study will provide further insights into the diversity of intrusive thoughts, which cannot be fully explained by OCD. Using co-clustering, we can effectively distinguish unique factors shared across participants and those that are individual-specific, thereby providing a structured framework for understanding the complex interactions underlying intrusive thoughts. This approach overcomes limitations in previous studies that either focus solely on relationships between factors (i.e., scale clusters only) or describe them exclusively by individual disorders (i.e., participant subtypes only). Consequently, this will provide foundational knowledge for developing support tailored to specific psychological states associated with intrusive thoughts.

## 2 Materials and methods

### 2.1 Scale selection

To provide an overview of the mechanisms underlying intrusive thoughts, we conducted a literature search using various databases and the search terms “Intrusive thought” AND “Obsessive-Compulsive Disorder OR Obsession OR OCD”. This search yielded 58 articles from Web of Science and an additional six articles from CiNii, a Japanese literature search engine (as of June 2018). From these articles, 31 scales related to intrusive thoughts were extracted from 42 studies (**Supplementary Figures 1**, **2**). Following discussions between the first and second authors, who are both licensed clinical psychologists, these scales were categorized into six conceptual categories: “obsessive beliefs,” “stress states,” “thought suppression,” “evaluation of intrusive thoughts,” “coping strategies,” and “obsessive-compulsive tendencies” (see **Supplementary Figures 1**, **2**). We examined the relationship between these six concepts using the Japanese measures described in the following sections.

### 2.2 Measures

#### 2.2.1 Obsessive beliefs

The Japanese version of the Obsessive Beliefs Questionnaire-44 (OBQ-44) was used (20). The OBQ-44 consists of three factors: “responsibility/threat estimation,” “perfectionism/certainty,” and “importance/control of thoughts,” comprising 44 items. Each item is rated on a seven-point Likert scale.

#### 2.2.2 Stress states

The Psychological Stress Response Scale (SRS-18) was used (21). The SRS-18 includes three factors: “depression/anxiety,” “irritability/anger,” and “hopelessness,” comprising 18 items in total. Participants rated each item on a four-point scale based on their feelings or behaviors over the past 2–3 days.

#### 2.2.3 Thought suppression

The Japanese version of the White Bear Suppression Inventory (WBSI) was used (1). The WBSI includes three factors: “thought suppression,” “unwanted intrusive thoughts,” and “self-distraction,” comprising 15 items in total. Participants rated the extent to which each item was applied on a five-point scale.

#### 2.2.4 Evaluation of intrusive thoughts

The Japanese Version of the Ego Dystonicity Questionnaire (EDQ-J) was used (22). The EDQ-J includes four factors: “irrationality,” “inconsistency with morals,” “implications of thought for personality,” and “repugnance,” comprising 16 items in total. Participants rated the extent to which each item applied to their recent experiences with intrusive thoughts on a six-point scale.

#### 2.2.5 Coping strategies

Coping strategies were assessed using the Tri-axial Coping Scale 24 (TAC-24) (23). The TAC-24 consists of eight factors: “getting information,” “giving up,” “evading one’s responsibility,” “plan drafting,” “positive interpretation,” “avoidance-like thinking,” “distractive recreation,” and “catharsis,” comprising 24 items in total. Participants rated the applicability of each item to their experiences in difficult situations on a five-point scale.

#### 2.2.6 Obsessive-compulsive tendencies

The Japanese version of the Maudsley Obsessive-Compulsive Inventory was used (24). The MOCI includes four factors: “checking,” “cleanliness,” “indecisiveness,” and “doubt,” comprising 30 items in total. Each item is rated on a three-point scale, based on the extent to which it is representative of typical thoughts and feelings.

### 2.3 Sample and procedure

A cross-sectional online survey was conducted among 302 participants aged 20 years and older through Rakuten Insight Inc. The questionnaire included free-text responses describing recent intrusive thoughts as well as standardized scales such as the OBQ-44, SRS-18, WBSI, EDQ-J, MOCI, and TAC-24. Participants were informed that their responses would remain anonymous and that their participation would be voluntary. Participants were instructed to respond only if they agreed to participate. Of the 302 participants, four who did not provide free-text responses were excluded, resulting in a final sample of 298 individuals (159 men and 139 women, with a mean age of 44.4 years ± 12.0). This study was approved by the Ethics Committee on Research with Humans as Subjects at Waseda University (Approval No. 2018-107).

### 2.4 Analytical procedure

Standard subscale scores were calculated for (1) obsessive beliefs (OBQ-44) (2), stress states (SRS-18) (3), thought suppression (WBSI), and (4) evaluation of intrusive thoughts (EDQ-J). Co-clustering was then applied to classify the co-occurrence relationships among the factors contributing to intrusive thoughts and to categorize them into different participant subtypes.

**Figure 1:**
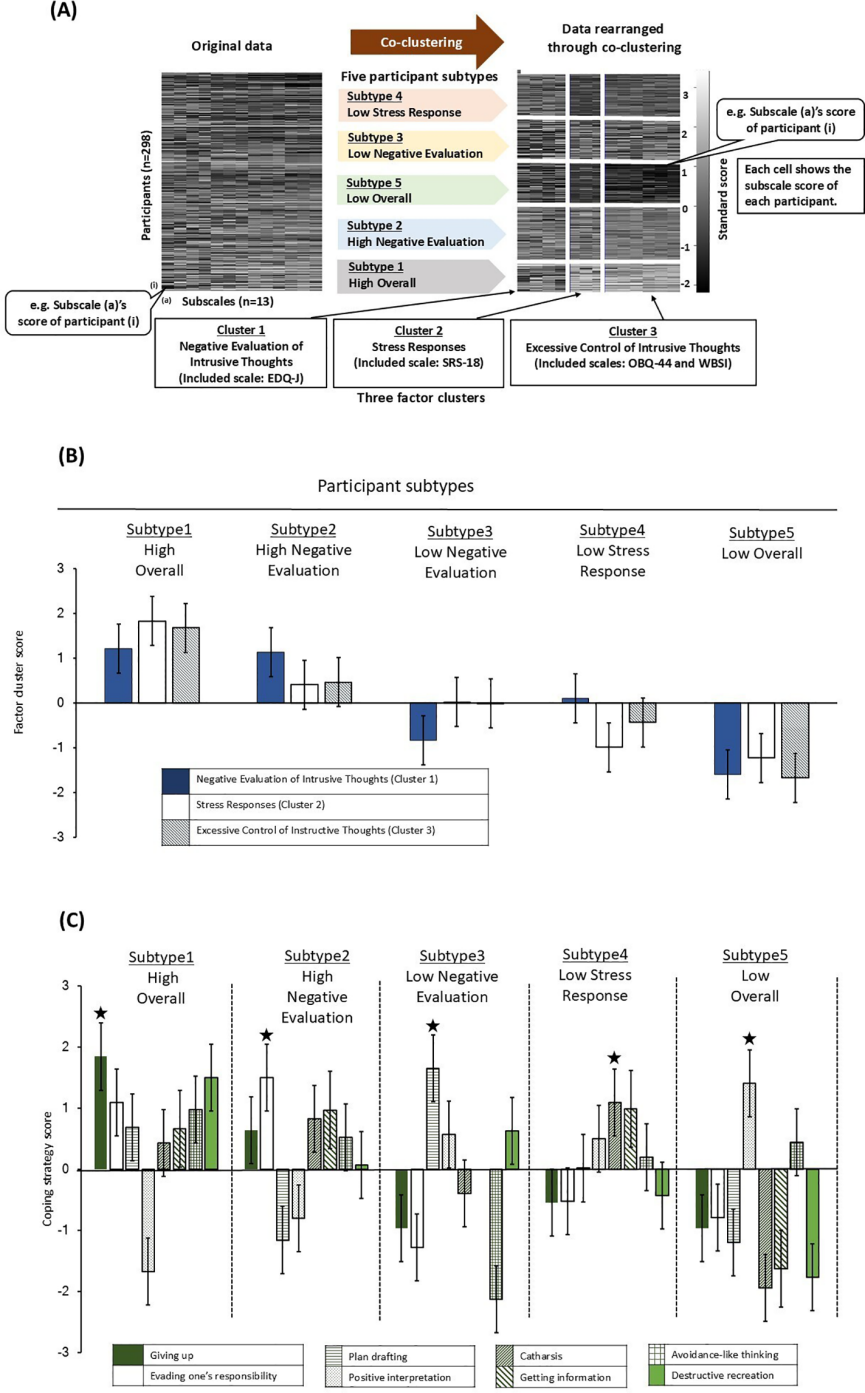
Co-clustering results and characteristics of each participant subtype. **(A)** Comparison of data distribution between original data and processed data by co-clustering. In raw data and data processed through co-clustering, each cell indicates the questionnaire standard score, with white and black color gradients indicating higher and lower scores (i.e., 3 – -2), respectively. In the data processed through co-clustering, rows indicate five subject subtypes and columns indicate three factorial clusters. 1) - 3) indicate cluster 1 – 3 and their titles, which we assigned what is represented; parentheses indicate subject scales used to categorize clusters. **(B)** Factor cluster scores of clusters 1 – 3 in five participant subtypes. Factor cluster scores regarding Cluster 1 – 3 (bar patterns corresponding to these clusters shown under the bar graph) in the indicated five subject subtypes are shown as bar graphs. Data are shown as mean values and vertical lines in the bars represent standard errors. **(C)** Coping strategy scores of five participant subtypes. We analyzed coping strategies used in the indicated five subject subtypes. Eight coping strategies corresponding to bar patterns are shown in the tables under bar graphs. Data are shown as mean values and vertical lines in the bars represent standard errors. (★) indicates the coping strategy with the highest score in each subject subtypes.

Co-clustering is a method that enables the simultaneous clustering of both rows and columns in data represented in matrix form while considering their relationships. For example, in the data obtained in this study, the columns represent participants, the rows represent scales, and each score in the matrix indicates the score of each participant on each scale (**Figure 1A**, left). In this way, it was possible to classify both the “co-occurrence relationships of intrusive thought factors (scale clusters)” and “participant state classifications (participant subtypes)” simultaneously, while considering their interrelations (**Figure 1A**, right). In this study, up to seven classifications for scale clusters and up to seven classifications for participant subtypes—resulting in a maximum of 49 data classifications—were permitted for co-clustering. Integrated Complete-data Likelihood (ICL) value was chosen as an indicator of goodness of fit for the co-clustering. The classification pattern with the highest ICL value was selected and interpreted (**Supplementary Figure 3**). However, classification patterns with fewer than 40 participants in any subtype were excluded for interpretability and their ICL values were not calculated. The choice of seven as the maximum number of classifications for both scales and participants was based on the fact that the goodness of fit for co-clustering tends to be higher when the classification aligns closely with the original data structure. Therefore, an upper limit was established in advance.

To understand the characteristics of each participant subtype, comparisons of the scores for the factors contributing to intrusive thoughts (scale cluster scores) (5), factors maintaining intrusive thoughts (TAC-24), and (6) obsessive-compulsive tendencies (MOCI) were conducted among participant subtypes using a one-way Analysis of Variance (ANOVA) followed by *post-hoc* comparisons. Additionally, one-sample t-tests were used to compare the scale cluster scores for factors contributing to intrusive thoughts across participant subtypes against the overall sample means. Bonferroni correction was applied to adjust p-values for multiple comparisons. The significance level was set at 5% and the trend significance level was set at 10% (25). Analyses were conducted using R (version 3.5.1), specifically employing the block-cluster package for co-clustering.

## 3 Results

### 3.1 Results of co-clustering

Standard scores for the subscales of participants’ obsessive beliefs (OBQ-44), stress state (SRS-18), thought suppression (WBSI), and evaluation of intrusive thoughts (EDQ-J) were calculated and co-clustering was performed. The scales were categorized into three clusters, and the participants were classified into five subtypes (**Figure 1A**; ICL value = -1163.3, pseudo-likelihood = -1025.4).

### 3.2 Interpretation of the classified factors of intrusive thoughts

An overview of the clusters obtained from co-clustering (**Figure 1A**, right) reveals that Cluster 1 includes the subscales of evaluation of intrusive thoughts (EDQ-J), Cluster 2 comprises subscales of stress state (SRS-18), and Cluster 3 contains the subscales of obsessive beliefs (OBQ-44) and thought suppression (WBSI) (**Table 1**). These results indicate that three distinct co-occurrence relationships regarding causative factors contribute to intrusive thoughts. Accordingly, Cluster 1 was named “Negative Evaluation of Intrusive Thoughts,” Cluster 2 as “Stress Responses,” and Cluster 3 as “Excessive Control of Intrusive Thoughts” as it appears to be related to the control of intrusive thoughts.

**Table 1:** Subscales included in each cluster.

Cluster	Scales included in the cluster	Subscales included in the cluster
[Cluster 1] Negative evaluation of intrusive thoughts	Evaluation of intrusive thoughts (EDQ-J)	“irrationality” “inconsistency with morals” “implications of thought for personality” “repugnance”
[Cluster 2] Stress response	Stress state (SRS-18)	“depression/anxiety” “irritability/anger” “hopelessness”
[Cluster3] Excessive control of intrusive thoughts	Obsessive beliefs (OBQ-44) and thought suppression (WBSI)	“responsibility/threat estimation” (OBQ-44 ) “perfectionism/certainty” (OBQ-44 ) “importance/control of thoughts” (OBQ-44 ) “thought suppression” (WBSI) “unwanted intrusive thoughts” (WBSI) “self-distraction” (WBSI)

### 3.3 Interpretation of the participant state classifications (participant subtypes)

#### 3.3.1 Differences in factors contributing to each participant subtypes

Participants were classified into five subtypes based on the differences in scoring patterns among the three clusters (**Figure 1A**, right). An overview of the results for each participant subtype, based on a series of statistical significance tests conducted in this study, is presented in **Table 2**.

**Table 2:** High/low/medium classification and coping strategies with the highest use scores among five participant subtypes.

	Items	Subject subtypes				
		Subtype 1 High Overall (n = 40)	Subtype 2 High Negative Evaluation (n = 77)	Subtype 3 Low Negative Evaluation (n = 59)	Subtype 4 Low Stress Response (n = 66)	Subtype 5 Low Overall Group (n = 56)
High/low/medium classification	Obsessive-compulsive tendencies	high	middle	middle	middle	low
	Negative evaluation of intrusive thoughts (Cluster 1)	high	high	low	middle	low
	Stress response (Cluster 2)	high	middle	middle	low	low
	Excessive control of intrusive thoughts (Cluster 3)	high	middle	middle	middle	low
	Coping strategies with the highest use scores	Giving up	Evading one’s responsibility	Plan drafting	Catharsis	Positive interpretation

Light gray (High) – high obsessive-compulsive tendencies, negative evaluation, stress responses, or control; Dark gray (Middle) – moderate level; No shading (Low) – low level.

To understand the characteristics of each subtype, one-sample t-tests were conducted to examine how each subtype’s scale cluster scores deviated from the overall mean value of the sample (**Figure 1B**). The results indicated that participants in Subtype 3 had significantly lower scores only in the “Negative Evaluation of Intrusive Thoughts” (*t* (76) = 6.12, *p* <.05), while Subtype 4 exhibited lower scores in both “Stress Responses” (*t* (65) = -13.26, *p* <.001) and “Excessive Control of Intrusive Thoughts” (*t* (65) = -7.79, *p* <.001) compared to the overall sample mean value. Subtypes 1 and 2 exhibited scores across all scale clusters that were higher than the overall mean value, whereas Subtype 5 demonstrated consistently lower scores than the mean value across all clusters (all *p*s <.001).

For further interpretation, scale cluster scores among subtypes were compared (**Figure 1B**). Significant main effects of subtype were observed for all scale cluster scores (**Table 3**, **Figure 1B**; “Negative Evaluation of Intrusive Thoughts,” *F* (4, 293) = 15.15, *p* <.001; “Stress Responses,” *F* (4, 293) = 109.3, *p* <.001; “Excessive Control of Intrusive Thoughts,” *F* (4, 293) = 251.5, *p* <.001). *Post-hoc* multiple comparisons revealed significant differences in “Negative Evaluation of Intrusive Thoughts” scores between Subtypes 1 and 3, 1 and 5, 2 and 3, 2 and 5, and 4 and 5 (all *p*s <.001), with an inversion observed in scores between Subtypes 3 and 4. Conversely, “Stress Responses” showed significant differences between Subtypes 2 and 3, and between 4 and 5, but not among the other subtypes (*p*(Subtype2-3) = .050, *p*(Subtype4-5) = .487, all other *p*s <.001), while significant differences were noted for “Excessive Control of Intrusive Thoughts,” across all subtypes (*p*(Subtype3-4) <.05, all other *p*s <.001), with no inversions in scores (**Table 3**).

**Table 3:** Comparison of cluster and coping strategy scores among five subject subtypes.

		Subtype 1	Subtype 2	Subtype 3	Subtype 4	Subtype 5	Main effects of groups	Multiple comparisons
		Mean values (SD)						
Cluster score	Obsessive-compulsive tendencies	75.10 (8.802)	70.83 (5.720)	67.29 (7.409)	66.00 (6.617)	59.89 (8.950)	*F*(4, 293)= 30.11, *p* <.001	(1)> (2)≒ (3)≒ (4)> (5)
	Cluster 1	0.467 (1.076)	0.431 (0.618)	-0.328 (1.217)	0.035 (0.673)	-0.621 (1.021)	*F*(4, 293)= 15.15, *p* <.001	(1)≒ (2)> (4)≒ (3)> (5)
	Cluster 2	1.502 (0.778)	0.392 (0.679)	0.089 (0.826)	-0.697 (0.427)	-0.885 (0.416)	*F*(4, 293)= 109.30, *p* <.001	(1)> (2)≒ (3)> (4)≒ (5)
	Cluster 3	1.531 (0.650)	0.423 (0.274)	0.141 (0.662)	-0.338 (0.351)	-1.426 (0.454)	*F*(4, 293)= 251.50, *p* <.001	(1)> (2)> (3)> (4)> (5)
Coping strategy score	Giving up	0.573 (1.120)	0.217 (0.831)	-0.265 (0.981)	-0.138 (0.819)	-0.266 (1.136)	*F*(4, 293)= 7.04, *p* <.001	(1)> (4) (1),>(3), (1)>(5), (2)>(3), (2)>(5)
	Evading one’s responsibility	0.283 (1.021)	0.387 (0.962)	-0.340 (0.880)	-0.141 (0.814)	-0.210 (1.157)	*F*(4, 293)= 6.80, *p* <.001	(2)>(1), (2)>(5), (2)>(3)
	Plan drafting	0.068 (1.043)	-0.090 (0.885)	0.150 (1.173)	0.009 (0.748)	-0.094 (1.186)	*F*(4, 293)= 0.66, *p* = .624	n.s.
	Positive interpretation	-0.341 (1.024)	-0.167 (0.865)	0.100 (1.064)	0.089 (0.937)	0.264 (1.093)	*F*(4, 293)= 3.02, *p* = .018	(5)>(1)
	Catharsis	0.057 (1.169)	0.120 (0.902)	-0.073 (1.089)	0.162 (0.888)	-0.319 (0.990)	*F*(4, 293)= 2.28, *p* = .061	(2)>(5), (4)>(5)
	Getting information	0.104 (1.244)	0.156 (0.846)	-0.179 (1.051)	0.160 (0.900)	-0.289 (1.008)	*F*(4, 293)= 2.69, *p* = .030	(2)>(5)
	Avoidance-like thinking	0.088 (1.064)	0.050 (0.917)	-0.186 (1.063)	0.019 (0.805)	0.042 (1.203)	*F*(4, 293)= 0.66, *p* = .621	n.s.
	Distractive recreation	0.229 (1.166)	0.022 (0.897)	0.105 (1.007)	-0.049 (0.792)	-0.247 (1.191)	*F*(4, 293)= 1.60, *p* = .175	n.s.
	Highest coping strategies	Giving up	Evading one’s responsibility	Plan drafting	Catharsis	Positive interpretation		

(>), significant difference or significant trend; (≒), no significant difference or trend; (n.s.), no significant difference. The significance level (<5%) and the trend (<10%). Values represent the mean (standard deviation). Scores for obsessive-compulsive tendencies are original scores; other scores are z scores.

Consequently, regarding the three factors contributing to intrusive thoughts (i.e., “Negative Evaluation of Intrusive Thoughts,” “Stress Responses,” and “Excessive Control of Intrusive Thoughts”), Subtype 1 exhibited high scores across all measures, Subtype 2 had high scores only in “Negative Evaluation of Intrusive Thoughts,” Subtype 3 showed low scores only in “Negative Evaluation of Intrusive Thoughts,” Subtype 4 had low scores only in “Stress Responses,” and Subtype 5 had low scores across all measures (**Figure 1B**). Based on these scoring patterns, the subtypes were labeled as “High Overall Group” (Subtype 1), “High Negative Evaluation Group” (Subtype 2), “Low Negative Evaluation Group” (Subtype 3), “Low Stress Response Group” (Subtype 4), and “Low Overall Group” (Subtype 5).

#### 3.3.2 Differences in maintaining factors among participants state classifications (participant subtypes)

Next, we examined the daily stress-coping strategies for each participant subtype. A distinctive result was observed: the highest-scoring coping strategy varied across participant subtypes. Specifically, the “High Overall Group” (Subtype 1) utilized “giving up,” the “High Negative Evaluation Group” (Subtype 2) relied on “evading one’s responsibility,” the “Low Negative Evaluation Group” (Subtype 3) favored “plan drafting,” the “Low Stress Response Group” (Subtype 4) used “catharsis,” and the “Low Overall Group” (Subtype 5) used “positive interpretation” as their most effective coping strategy (marked with ★ in **Figure 1C**).

Further investigation showed that significant main effects were observed for “getting information,” “giving up,” “evading one’s responsibility,” and “positive interpretation,” while a trend toward significance was observed for “catharsis.” Specifically, the “High Overall Group” demonstrated significantly higher scores for “giving up.” The “High Negative Evaluation Group” exhibited a significantly higher score for “evading one’s responsibility,” The “Low Negative Evaluation Group” displayed significantly higher scores for “catharsis.” and the “Low Overall Group” showed significantly higher scores for “positive interpretation” compared to at least one other participant subtypes (**Table 3**). However, no significant trend was observed for “planning” within the “Low Negative Evaluation Group,” when compared to the other participant subtypes (**Table 3**).

Overall, these findings suggest that the coping strategies used by each participant subtype differ and that the factors that maintain intrusive thoughts are unique to each subtype.

#### 3.3.3 Differences in the degree of obsessive-compulsive tendencies among participants state classifications (participant subtypes)

Finally, we calculated the degree of obsessive-compulsive tendencies (MOCI scores) for each subtype and examined the differences in obsessive-compulsive tendencies among the participant subtypes. The results indicated a significant main effect for subtype (*F* (4, 293) = 30.11, *p* <.001). *Post-hoc* comparisons revealed that both the “High Overall Group” and “Low Overall Group” (Subtypes 1 and 5) had significantly higher or lower scores than all other subtypes (all *p*s <.05). In contrast, the “Low Negative Evaluation Group” (Subtype 3) exhibited obsessive-compulsive tendency levels comparable to those of the “High Negative Evaluation Group” (Subtype 2) and “Low Stress Response Group” (Subtype 4) (*p*(Subtype2-3) = .059, *p*(Subtype3-4) = 1.000).

In summary, the participant subtypes can be understood as follows: the “High Overall Group” (Subtype 1) exhibited a high degree of obsessive-compulsive tendencies, the “High Negative Evaluation Group” (Subtype 2), “Low Negative Evaluation Group” (Subtype 3), and “Low Stress Response Group” (Subtype 4) demonstrated moderate levels, while the “Low Overall Group” (Subtype 5) showed low levels of obsessive-compulsive tendencies (**Table 2**).

## 4 Discussion

In this study, we examined the differences in patterns of intrusive thoughts based on a classification of the factors contributing to the occurrence of intrusive thoughts. Through the co-clustering analysis, we found that three key factors (i.e., “Negative Evaluation of Intrusive Thoughts,” “Stress Responses,” and “Excessive Control of Intrusive Thoughts”) are involved in the occurrence of intrusive thoughts. Furthermore, differences in scoring patterns among these factors allowed us to classify intrusive thoughts into five distinct subtypes. Specifically, the “High Overall Group” (Subtype 1) and “Low Overall Group” (Subtype 5) were identified, confirming that they corresponded to participants with high and low levels of obsessive-compulsive tendencies, respectively. Conversely, participants with moderate obsessive-compulsive tendencies were classified into three subtypes: “High Negative Evaluation Group” (Subtype 2), “Low Negative Evaluation Group” (Subtype 3), and “Low Stress Response Group” (Subtype 4). These findings suggest that intrusive thoughts cannot be fully explained by the degree of obsessive-compulsive tendencies, and may provide important insights various states of intrusive thoughts not limited to OCD.

Notably, by comparing the “High Negative Evaluation Group” (Subtype 2), “Low Negative Evaluation Group” (Subtype 3), and “Low Stress Response Group” (Subtype 4), it became evident that the occurrence of intrusive thoughts could not be explained solely by the severity of obsessive-compulsive tendencies. For instance, when comparing the “High Negative Evaluation Group” (Subtype 2) and the “Low Negative Evaluation Group” (Subtype 3), the primary difference lay in the degree of “Negative Evaluation of Intrusive Thoughts,” This suggests that while “Negative Evaluation of Intrusive Thoughts” contribute to their occurrence, it does not necessarily correlate with the severity of obsessive-compulsive tendencies. In fact, intrusive thoughts have been documented in other disorders such as substance use disorders, depression, post-traumatic stress disorder, and anxiety disorders, where the negative evaluation of thoughts as ego-dystonic is understood as a common pathology (24). Thus, intrusive thoughts may be explained by factors independent of OCD, which may account for the observation of intrusive thoughts in diverse populations.

Similarly, by comparing the “Low Negative Evaluation Group” (Subtype 3) and “Low Stress Response Group” (Subtype 4), the complex interactions of the factors contributing to the occurrence of intrusive thoughts were further confirmed. Given that “Negative Evaluation of Intrusive Thoughts” is independent of obsessive-compulsive tendencies, the “Low Stress Response Group” (Subtype 4) was predicted to have lower obsessive-compulsive scores than the “Low Negative Evaluation Group” (Subtype 3). This is because the “Low Stress Response Group” (Subtype 4) showed opposite scores for “Negative Evaluation of Intrusive Thoughts” and “Stress Responses” compared to the “Low Negative Evaluation Group” (Subtype 3), while displaying “Stress Responses” similar to those of the “Low Overall Group” (Subtype 5). However, as mentioned previously, no significant differences were found in the severity of obsessive-compulsive tendencies between the two groups. These findings suggested that “Stress Responses” do not directly influence the severity of obsessive-compulsive tendencies, but rather has an indirect effect on these obsessive-compulsive tendencies influencing the levels of other factors. Previous research has indicated that stress responses serve as moderating factors that control the severity of intrusive thoughts (23). The present results suggest that intrusive thoughts should be considered based on the variations in subtypes, which are characterized by interrelationships among multiple factors.

Finally, when comparing the “High Negative Evaluation Group” (Subtype 2) and the “Low Stress Response Group” (Subtype 4), differences were found in scores for “Stress Responses,” and “Excessive Control of Intrusive Thoughts,” as well as in the obsessive-compulsive tendencies. This suggests that the higher the scores for “Stress Responses,” and “Excessive Control of Intrusive Thoughts,” the stronger the obsessive-compulsive tendencies become. These tendencies appear to be consistent across all participant subtypes. Therefore, these results suggest that intrusive thoughts occur from the interaction between the cognitive factor of “Excessive Control of Intrusive Thoughts” and the environmental factor of “Stress Responses,” providing a theoretical basis to explain the continuity of intrusive thoughts between OCD patients and healthy individuals. Indeed, previous studies have pointed out that in patients with OCD, higher levels of perceived stress increase intrusive thoughts, and attempts to control intrusive thoughts paradoxically lead to their increase (16, 26).

However, the persistence of intrusive thoughts has been examined in previous studies from the perspective of interactions with daily behaviors such as “stress-coping strategies” (27, 28). Therefore, in this study, we also examined the differences in coping strategies among participant subtypes and, as a result, more clearly demonstrated the diversity in intrusive thoughts. Specifically, each participant subtype used different coping strategies: the “High Overall Group” (Subtype 1) tended to use “giving up,” the “High Negative Evaluation Group” (Subtype 2) “evading one’s responsibility,”‘ the “Low Negative Evaluation Group” (Subtype 3) “plan drafting,” the “Low Stress Response Group” (Subtype 4) “catharsis,” and the “Low Overall Group” (Subtype 5) “positive interpretation.” According to the interpretation of TAC-24, “giving up” is classified as “avoidance/problem focus/cognitive type,” “evading one’s responsibility”‘ as “avoidance/problem focus/behavioral type,” “plan drafting” as “encounter/problem focus/cognitive type,” “catharsis” as “encounter/emotion focus/behavioral type,” and “positive interpretation” as “encounter/emotion focused/cognitive type (29).” Therefore, a moderate relationship was observed, indicating that participant subtypes with higher scores on “Stress Responses,” and “Excessive Control of Intrusive Thoughts” tend to employ more avoidant and problem-focused coping strategies. However, the specific coping strategies unique to each participant subtype could not be predicted based on factor scores related to the occurrence of intrusive thoughts. Therefore, classifying participant subtypes using co-clustering was particularly useful for examining the complex interactions between factors associated with both the occurrence and persistence of intrusive thoughts. This highlights the added value of the co-clustering approach, which enabled simultaneous classification of participant subtypes and scale clusters. By considering their interactions, co-clustering revealed unique coping strategies characteristic of each subtype, providing insights that traditional scale classification methods could not obtain.

The co-clustering approach provides a valuable framework for personalizing treatments and advancing our understanding of the mechanisms underlying intrusive thoughts. Our findings underscore the necessity of personalized approaches in understanding and addressing intrusive thoughts. For example, the “High Negative Evaluation Group” (Subtype 2) exhibited high scores on “Negative Evaluation of Intrusive Thoughts”. Cognitive interventions should be prioritized for this group, with additional adjustments made according to individual needs. Meanwhile, the “Low Negative Evaluation Group” (Subtype 3), characterized by heightened stress responses and control of intrusive thoughts, may benefit from stress management programs alongside cognitive-behavioral therapy. On the other hand, individuals in Subtype 4 have low stress levels but high negative evaluations and excessive control of intrusive thoughts. This group might benefit from mindfulness interventions that train them to distance themselves from their thoughts, thereby fundamentally changing the way they interact with their thoughts. Additionally, given the interactions between cognitive and environmental factors observed in this study, daily coping strategies are likely to evolve with appropriate interventions. This highlights the importance of incorporating intervention strategies that address both situational and individual factors.

This study has several limitations. First, the participants state classifications and interpretations adopted in this study are not the only possibilities. If other scales were added to those used in this study, or if the scales analyzed through co-clustering changed, different results could be obtained. However, the scales used in this study were carefully selected to avoid conceptual overlap based on the literature review, and the attempt to divide them into factors associated with the occurrence of intrusive thoughts and factors associated with persistence of intrusive thoughts was grounded in previous studies. The interpretation of data-driven analysis results is a general issue when applying machine-learning methods, and this should continue to be discussed in the future. Second, as is evident from the score relationships of the factors associated with the occurrence of intrusive thoughts, a moderate correlation was between participant subtypes. These results suggest that understanding intrusive thoughts from a trait-based perspective is more important than from a typological perspective. Therefore, we avoided treating each participant subtype as qualitatively distinct, which limited our ability to compare the subtypes. Despite the limitations of this discussion, our findings provide strong evidence that the psychological states associated with intrusive thoughts are not homogeneous and are significantly diverse. This issue can be addressed by expanding the scale and exploring a wider range of factors related to intrusive thoughts. Third, the selection of the number of clusters in this study, while guided by goodness-of-fit indicators, involves a certain degree of arbitrariness. In the field of machine learning, techniques such as cross-validation have been used to examine the accuracy of classification and number of clusters. This study did not conduct validation of clustering accuracy in line with existing research in the clinical field that uses clustering. However, future studies should examine the accuracy of clustering by increasing the amount of available data. Fourth, there was a limitation in that the alignment of scale clusters with the original subscale classifications appeared to restrict novelty. However, the co-clustering approach provided unique value by uncovering complex interactions between factors related to the occurrence and persistence of intrusive thoughts. This approach enabled us to identify participant clustering results and coping strategies that would not have been visible through conventional classification methods. Fifth, although this study suggests a relationship between intrusive thoughts and obsessive-compulsive tendencies, the absence of detailed assessments of anxiety and depression levels limits our ability to account for potential confounding effects. Including appropriate measures in future studies could further clarify the relationships between these psychological states and intrusive thoughts. For example, scales specific to depressive or anxiety-related intrusive thoughts might reveal additional clusters or more nuanced patterns of interaction between factors. This remains an important direction for future research.

Despite these limitations, this study highlights the differences in the patterns of factors influencing the occurrence of intrusive thoughts, emphasizing the diversity of psychological state differences in intrusive thoughts, which have previously been examined primarily as symptoms of OCD. Notably, we found that coping strategies for intrusive thoughts cannot be fully explained by OCD tendencies alone. Therefore, to approach these coping strategies, it is crucial to identify and understand the complex interactions among the factors contributing to the occurrence of intrusive thoughts that influence these coping strategies. These finding provides robust support for the hypotheses of continuity and diversity in intrusive thought from a data-driven perspective and provides a clear explanation as to why these thoughts are not disease-specific, which had not been clarified in previous research. In the future, more comprehensive assessments of the scale related to occurrence and persistence factors should be conducted with a larger population; by performing big data analysis, clearer and more fully interpretable characteristics of intrusive thoughts will be elucidated.

## Data Availability

The raw data supporting the conclusions of this article will be made available by the authors, without undue reservation.
